# Histological transformation in recurrent WHO grade I meningiomas

**DOI:** 10.1038/s41598-020-68177-x

**Published:** 2020-07-08

**Authors:** Marco V. Corniola, Jean-Michel Lemée, Torstein R. Meling

**Affiliations:** 1 Department of Neurosurgery, Geneva University Hospitals, Rue Gabrielle Perret Gentil 4, 1205 Geneva, Switzerland; 2 Department of Neurosurgery, Angers University Hospitals, Rue Larrey 4, 49100 Angers, France

**Keywords:** Surgical oncology, Cellular neuroscience, Risk factors, Epidemiology

## Abstract

Atypical or malignant transformation (AT/MT) has been described in WHO grade I meningiomas. Our aim was to identify predictive factors of AT/MT at recurrence. A total of N = 15 WHO grade increases were observed in N = 13 patients (0.96% of the study population, risk of transformation of 0.12% per patient-year follow-up). Patients with and without progression at recurrence were similar regarding age, gender distribution, skull-base location, bone infiltration, and Simpson grades. Recurrence-free survival was lower in patients with transformation (5 ± 4.06 years versus 7.3 ± 5.4 years; p = 0.03). Among patient age, gender, skull base location, extent of resection or post-operative RT, no predictor of AT/MT was identified, despite a follow-up of 10,524 patient-years. The annual risk of transformation of WHO grade I meningiomas was 0.12% per patient-year follow-up. Despite the important number of patients included and their extended follow-up, we did not identify any risk factor for transformation. A total of 1,352 patients with surgically managed WHO grade I meningioma from a mixed retro-and prospective database with mean follow-up of 9.2 years ± 5.7 years (0.3–20.9 years) were reviewed. Recurring tumors at the site of initial surgery were considered as recurrence.

## Introduction

Meningiomas are the most frequent intracranial, extra-cerebral tumors and represent up to 40% of primary intracranial tumors in surgical series^1,2^. Whilst their etiology is still not fully understood, it is known that genetic inherited dispositions, hormonal effects, ionizing radiation exposure, as well as immunological factors are implicated in their genesis^3,4^.

According to the latest recommendations^5^, the management of meningiomas is primarily surgical and aims for a complete/maximal resection of the tumor, including its dural tail. A meningioma management according to the highest standards of care eventually offers high rates of disease-control and globally favorable outcomes^1,6–9^.

The European Association of Neuro-Oncology (EANO) guidelines^5^ advises a close and diligent post-operative clinico-radiological follow-up, as even long-term recurrences may occur^10^. The 14 monosomy and 1p36 chromosomal deletion^11^, the location of the meningioma^1,11^. a sub-total resection (STR)^1,8,11^ and higher World Health Organization (WHO) grade^1,12,13^ are amongst the patient-, tumor- and surgery-related factors of recurrence identified and described in the literature.

The grading of the meningiomas is based on the WHO classification, ranging from grade I to III, with respective overall and progression-free survivals (OS & PFS)^5,14^. Although up to 3% are malignant (WHO grade III)^5,15,16^ and 5–7% atypical (WHO grade II), the vast majority of meningiomas are benign (WHO grade I) and show an indolent progression^6^. Therefore, meningiomas can be perceived as a chronic disease in most of the cases^10,17^.

However, similarly to what can occur in low-grade gliomas (LGG), malignant transformation (MT) has previously been described in formally identified WHO grade I meningiomas^18–21^. The overall rate of progression from benign to non-benign histology ranges from 0.16% to 2.0%, according to the most recent data available in the literature^22–25^. In the case of LGGs, risk factors for malignant transformation (age, multiple tumor location, management by chemotherapy alone and STR) have been described^25^. Similarly, age and non skull-base location has been suggested as predictors for atypical or malignant transformation (AT/MT) at recurrence in meningiomas^9,15,26–28^. This statement is of paramount importance, since patients with recurring meningioma with AT/MT may need more aggressive treatment. Therefore, it is primordial that neurosurgeons and neuro-oncologists are able to identify properly those patients at risk for AT/MT.

In this study, a large cohort of patients with recurring WHO grade I meningiomas is presented, focusing on the progression toward higher WHO grades at recurrence. Our aim is to identify the predictive factors of AT/MT at recurrence, using a large dataset with an exhaustive and very long follow-up, representing a total of 10, 524 patient-years.

## Results

### WHO grade increase in recurrent meningiomas and population characteristics

Baseline demographics, clinical and radiological characteristics of the study cohort are summarized in Table 1. A total of N = 15 WHO grade increases at recurrence were observed in N = 13 patients, representing 0.96% of the study population and corresponding to a risk of transformation of 0.12% per patient-year follow-up. Patients with and without progression at recurrence were similar regarding age, gender distribution, skull-base location and bone infiltration (Table 1). The Simpson grades were also similar between the two groups.

**Table 1:** Baseline clinico-radiological data, overall survival and progression-free survival of patients with and without WHO grade increase at first recurrence.

	WHO grade increase		No WHO grade increase		p
	n = 13	%	n = 1,342	%	
**Age**	55.5 ± 11.6	–	58 ± 13.2	–	0.44
**Sex**	8 F/5 M	–	957 F/382 M	–	0.53
**Skull base tumor**	5	38.5	653	48.7	0.58
**Bone infiltration**	1	7.7	248	18.5	0.48
**Simpson grade after initial surgery**					0.28
I	4	30.8	532	39.8	
II	6	46.2	458	34.2	
III	0	0	70	5.3	
IV	3	23	272	20.4	
V	0	0	5	0.3	
**Postop RT after 1st surgery**	0	0	28	2.1	0.96
**OS**	11.1 ± 6.2	–	9.2 ± 5.6	–	0.29
**RFS**	5 ± 4.1	–	7.3 ± 5.4	–	0.03

*OS* overall survival, *Neuro. Worsening* neurological worsening, *Postop RT* post-operative radiation therapy, *PFS* progression-free survival.

The distributions of WHO grades at second and third surgery are shown in Table 2. A total of N = 11 transformations were observed between the first and the second surgery; 9 increases to WHO grade II and 2 increases to WHO grade III. At the third surgery, N = 4 new transformations were observed: N = 2 from WHO grade I to WHO grade II and N = 2 from WHO I already transformed in grade II to WHO grade III. Two patients were operated 3 times with an increase of WHO grade at each surgery.

**Table 2:** Distribution of the WHO grades at the second and the third surgery in originally WHO grade I meningiomas.

WHO grade	2nd surgery	3rd surgery
I	76 (87.4%)	14 (70%)
II	8 (9.2%)	3 (15%)
III	3 (3.4%)	3 (15%)
Total	87	20

In the WHO grade progression group, no patient had post-operative radiation therapy (RT). In the group with WHO grade stability, a total of N = 28 patients had an adjuvant RT after the first surgery. No difference was observed between the groups, as shown in Table 1.

### Retreatment-free survival

**Figure 1:**
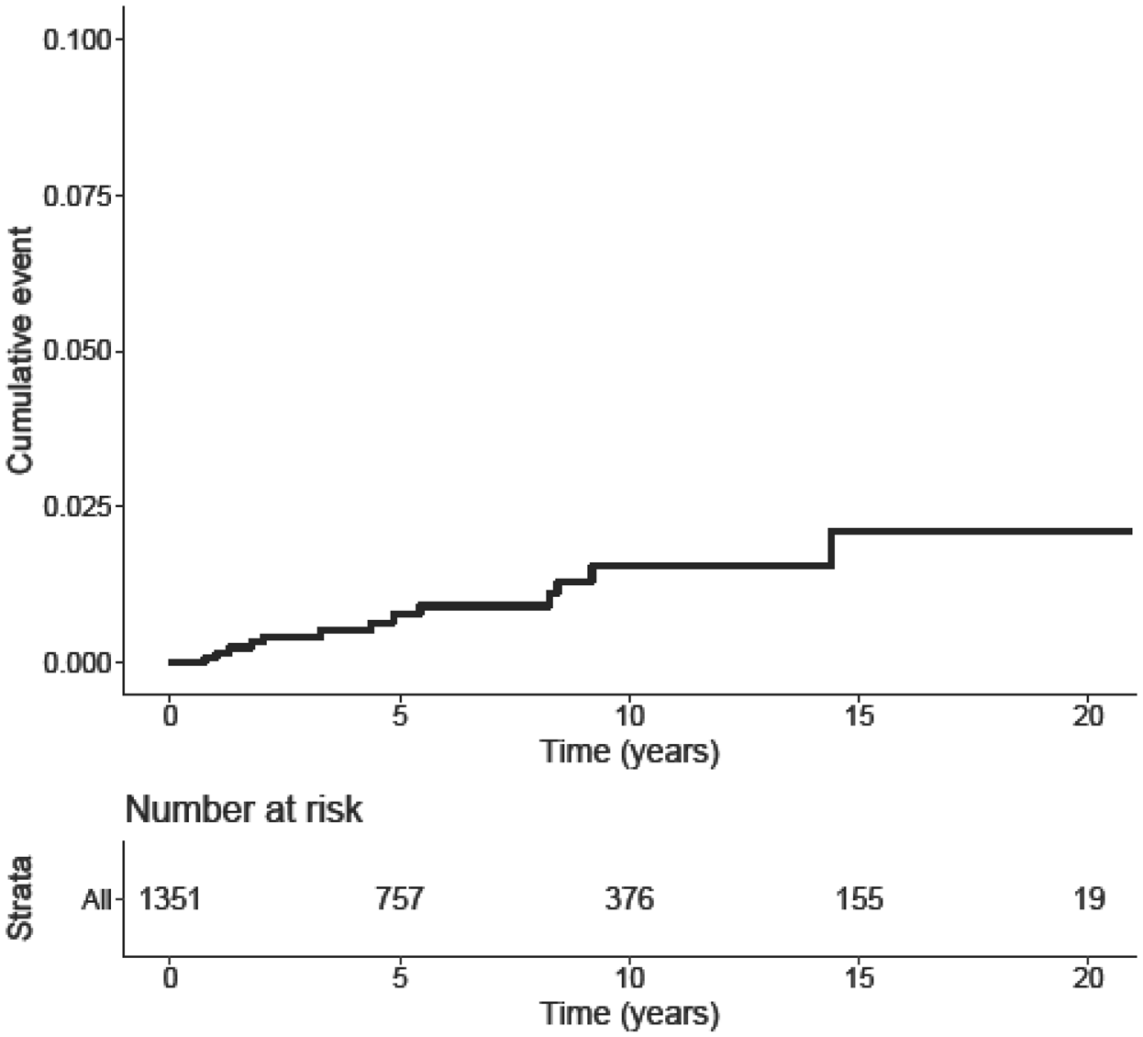
Kaplan–Meier curve of progression in WHO grade over time, with the respective number at risk.

**Figure 2:**
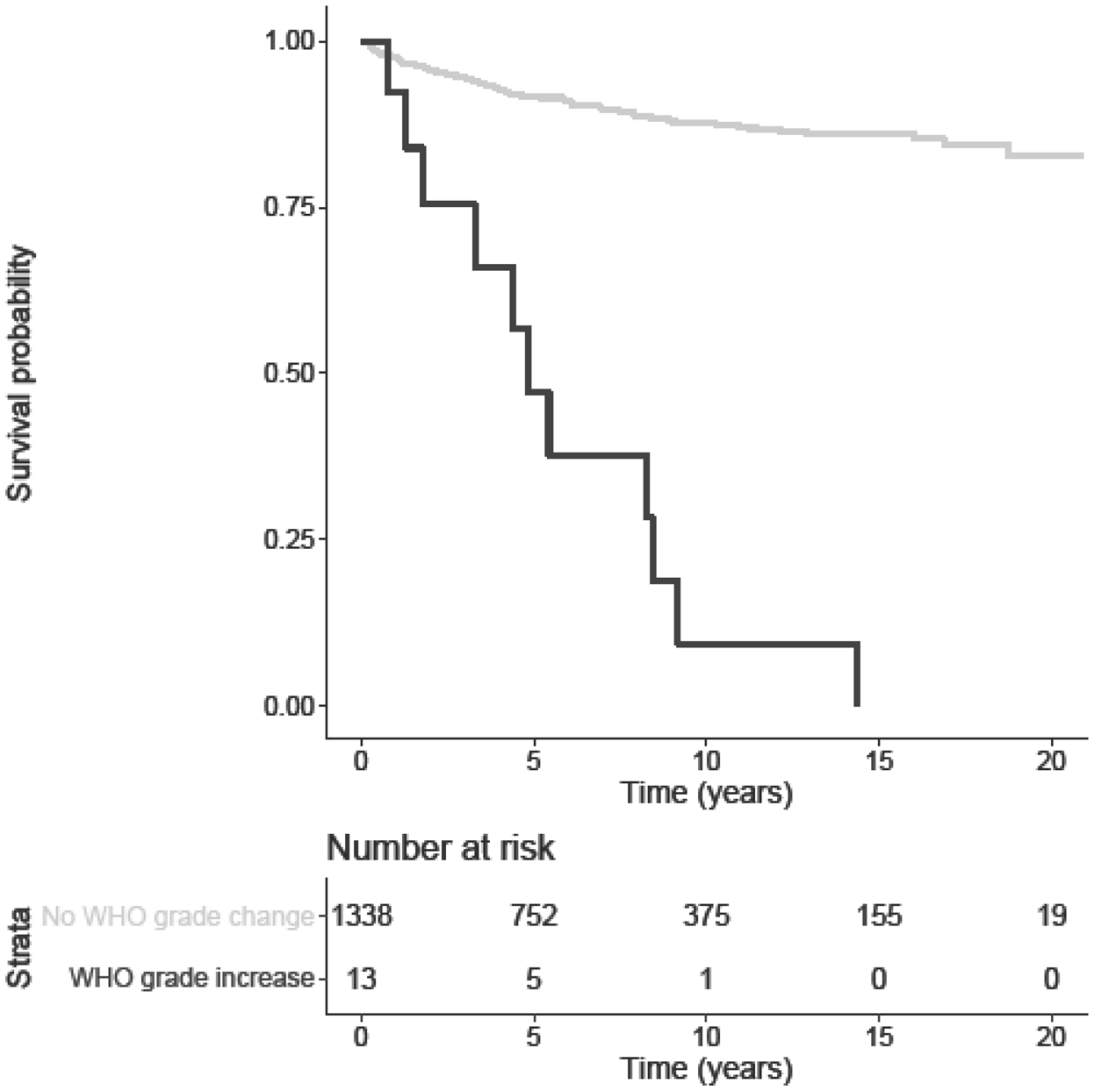
Kaplan–Meier curves of PFS in patients with (dark grey) and without (light grey) WHO grade change.

Data on RFS and OS are shown in Table 1. RFS was significantly lower in patients with transformation when compared to patients without transformation (5.0 ± 4.1 years versus 7.3 ± 5.4 years; p = 0.03). The mean RFS in tumors showing a progression of one WHO grade was 4.8 ± 4.1 years, whereas the mean RFS in tumors showing a progression of two WHO grades was 8.1 ± 8.9 years. Two patients presented outlying time-to-progression (1.8 years and 14.4 years). Interestingly, the OS did not significantly differ between the two cohorts. The Kaplan–Meier curve of WHO grade progression probability over time is shown in Fig. 1. The Kaplan–Meier curves of PFS in progressing and non-progressing recurrences are shown in Fig. 2.

### Predictive factors of WHO grade transformation

No predictive factor for WHO grade progression at recurrence was found amongst the age, gender, skull base location, the extent of resection as well as the post-operative RT, as shown in Table 3.

**Table 3:** Predictive factors of WHO grade increase after first surgery.

	WHO grade increase	
	Odd ratio	p
Age	0.98 [0.94; 1.02]	0.43
Sex (male)	1.54 [0.46; 4.69]	0.46
Skull base location	0.71 [0.21; 2.18]	0.56
Simpson grade of first surgery	1.05 [0.63; 1.66]	0.84
RT after first surgery	0.10 [0.01; 5.06]	0.99

*RT* radiation therapy.

## Discussion

In this study, we in investigated the risk of atypical or malignant transformation (AT/MT) in recurring intracranial WHO grade I meningiomas, using a database of 1,352 patients treated by surgery with a cumulated total of 10,524 patient-years follow-up. The annual risk of AT/MT was 0.12% per patient-year follow-up, representing approximately 1% of surgically managed patients, and 19.5% of the patients treated for a recurrence.

Previous studies have identified advanced age and non-skull base location as predictors for AT/MT in recurring meningiomas^15,29^. Despite the large number of patients included and their long and complete follow-up, no risk factor for transformation among gender, skull-base location, Simpson grade after first surgery, radiotherapy after initial surgery, and advanced age could be identified. We therefore conclude that should risk factors of AT/MT exist, their effect is probably minor.

According to the latest EANO recommendations, the post-operative follow-up of meningiomas should be performed by a senior neurosurgeon; the interval between follow-up visits can vary considerably, depending on the Simpson grade, the initial size of the lesion, its location, the age of the patient, as well as its general neurological condition^5^. In the case of WHO grade I meningiomas with documented GTR, the recurrence rate at 10 year ranges from 20 to 39%^7,30,31^. Therefore, an annual follow-up is recommended, up to 5 years after the treatment, then every 2 years. Our results corroborate these data. However, one of our patients had a meningioma recurrence with AT/MT increase more than ten years after initial diagnosis. According to the literature, this is no exception; in their long-term follow-up of surgically managed parasagittal meningiomas, Petersson-Segerlind et al. stated that the 25 years recurrence rate was up to 47%. More specifically, the 10- and 25-years recurrence rates for Simpson grade I–II resections of parasagittal meningiomas were 13% and 48%, respectively. The authors found that the 10- and 25-years mortality rates were as high as 33% and 63%, respectively, of which 50% and 48% of the mortality were directly attributable to the tumor at 10 and 25 years, respectively^10^. Data on incidentally, observed meningiomas can support these findings. Jadid et al. followed a cohort of consecutive patients referred with incidentally discovered, asymptomatic meningiomas for at least 10 years and found that 35.4% of the tumors showed growth (regardless of tumor size), resulting in a 75% 15-year growth-rate by life-table statistics^17^.

To our knowledge, this is the first population-based study assessing AT/MT in originally and formally identified WHO grade I meningiomas using a large cohort with a very long and complete follow-up. Our findings are of paramount importance for neurosurgeons, as they should be able to estimate and inform the patient of the rate of atypical or malignant transformation of meningiomas, that may need an earlier second surgery, but without impact on OS.

### Strengths and limitations

Our dataset offers a comprehensive and very extended source of data on patients with intra-cranial meningiomas with complete follow-up, since only one patient was lost during the study period.

The small number of histological transformations (N = 15 WHO increases in N = 13 patients) limits the finding of statistically associated prognostic factors. However, our cohort is one of the largest, to our knowledge. As WHO classification changed several times over the duration of the study, re-classification of the tumors carries risk of mis-grading, also representing a limitation.

The main limitation is the missing of data on molecular characteristics (MIB-1), because of the extended period of inclusion and could not be retrieved for the analysis. The importance of genetics and biomarkers such as MIB-1 and Ki-67 is very well established in the management of meningiomas, at first surgery as well as whenever recurrence occurs^15,32,33^. For instance, McGovern et al.^15^ found that WHO grade I non-skull base meningiomas have a higher MIB-1 index than grade I skull base meningiomas (1.77 ± 1.86% versus 3.44 ± 3.53%), correlating with lower RFS. Matsuno et al.^32^ assessed the proliferative potentials of meningiomas using the anti-Ki-67 monoclonal antibody and the MIB-1. The authors observed a statistically significant difference in the MIB-1 index related to gender and age as male patients showed a mean index of 5.5%, versus 2.7% in female patients, and higher MIB-1 indexes were observed in younger patients. Meningiomas with a MIB-1 index ≥ 3% were found to have a significantly high tendency for recurrence within the first 10-year follow-up periods. These results were corroborated by Perry et al.^33^ Unfortunately, were not able to retrieve information about MIB-1 status of the tumor, as these data were not available for the whole cohort, due to the extended time of recruitment. Certainly, the future will open new perspectives in molecular diagnosis in meningioma management, including the development of biomarkers for AT/MT.

Lastly, analysis of the methylation profile has recently been developed and reported by Sahm et al.^34^ It is a new yet powerful method for analyzing epigenetic changes in tumor DNA. The analysis of methylation is increasingly used in CNS tumors overall, with excellent diagnostic and prognosis values in terms of biological behavior, open new perspectives towards the identification of predictors of grade changes in initially WHO grade I recurring meningiomas, for which patient-, tumor- and surgery-related characteristics are not sufficient to identify predictors for AT/MT. Unfortunately, we were not able to retrieve information about methylation status of the tumor.

## Conclusion

The annual risk of transformation of WHO grade I meningiomas is 0.12% per patient-year follow-up. Despite the important number of patients included in the study and their extended follow-up, we were not able to identify any risk factor for transformation, among gender, skull-base location, advanced age, Simpson grade after first surgery and first postoperative RT.

## Methods

### Patient cohort

We performed a review of a cohort of intracranial meningiomas consecutively treated by surgery at Oslo University Hospital (OUH). OUH is a tertiary referral center including two neurosurgical units (Rikshospitalet and Ullevaal), covering a total of 3 million inhabitants (representing circa 56% of the Norwegian population). A total of 1,469 consecutive patients were identified from a mixed retro-and prospective database (from 1990 to 2002 and from 2003 to 2010, respectively). For this study, only patients with formally identified, surgically managed WHO grade I intracranial meningioma were considered, representing a total of 1,352 patients.

In most cases, surgery aimed at complete tumor resection, considering both patient and tumor characteristics. Based on surgical reports and the post-operative imaging studies, the extent of resection (EOR) was assessed using the Simpson grade scale^30^. Gross total resection (GTR) was defined as a Simpson grade I–III resection, according to the latest guidelines of the European Association of EANO^5^.

The histopathological diagnosis of meningioma was confirmed by a senior pathologist on a case-by-case basis. Since the WHO criteria changed during the study period, the tumors included between 1990 to 2001 were re-classified as WHO grade I, WHO grade II and WHO grade III by a neuropathologist (previously benign, atypical, and anaplastic, respectively).

The mean follow-up was 9.2 years ± 5.7 years (range 0.3–20.9 years), with a cumulated total of 10,524 patient-years follow-up. One patient was lost of follow-up due to moving abroad. Regarding the post-operative clinico-radiologic follow-up, patients were generally followed yearly or after 1 and 3 years and then every 5 years. However, the follow-up did not follow a rigorous policy since the initial size of the lesion, its location, the Simpson grading of the resection, the age of the patient and the neurological status were considered on a case-by-case basis.

Recurring tumors at the site of initial surgery with radio-clinical correlations were considered as recurrence. Retreatment-free survival (RFS or time-to-retreatment) was defined as the time between the first surgery and the second surgical procedure. Radiological recurrences without clinical impact not requiring any adjuvant treatment, as well as tumors occurring remotely to the primary site, were not considered as recurrence and thus not included in the analysis. In recurring tumors, histo-pathological data was recorded up to the third surgery. In cases where patients were operated more than three times, data on following surgeries were not included in the analysis. As a result, from a dataset comprising N = 1,352 meningiomas, a total of N = 87 and N = 20 patients with first and second recurrence were available, respectively.

### Ethics

The study was regulated by the Personal Data Act/Personal Health Data Filing System Act and approved by the Data Protection Official, registered Norwegian institutional review board at OUH (2017/5204). Under this regulation, patient’s written informed consent was not required to collect and analyze data. The study was performed in accordance with the ethical standards of the institutional and/or national research committee and with the 1964 Helsinki declaration and its later amendments or comparable ethical standards.

### Statistical analysis

The statistical analysis was performed using R v3.5.1 (https://www.r-project.org). The statistical significance threshold was set at p = 0.05. A binomial logistic regression following a general linear model approach was used to identify predictive factors for AT/MT at recurrence. A Kaplan–Meier approach was used to assess the risk of transformation over time.

## Data Availability

If requested by the editors, the authors will fully cooperate in obtaining and providing the data on which the manuscript is based without any restriction.
